# Small molecule modulation of the p75 neurotrophin receptor inhibits multiple amyloid beta-induced tau pathologies

**DOI:** 10.1038/s41598-020-77210-y

**Published:** 2020-11-23

**Authors:** Tao Yang, Kevin C. Tran, Anne Y. Zeng, Stephen M. Massa, Frank M. Longo

**Affiliations:** 1 Department of Neurology and Neurological Sciences, Stanford University School of Medicine, 300 Pasteur Drive, Room H3160, Stanford, CA 94305 USA; 2 Department of Neurology, San Francisco Veterans Affairs Health Care System, University of California, San Francisco, 4150 Clement St., San Francisco, CA 94121 USA

**Keywords:** Alzheimer's disease, Mechanism of action, Neurotrophic factors

## Abstract

Longitudinal preclinical and clinical studies suggest that Aβ drives neurite and synapse degeneration through an array of tau-dependent and independent mechanisms. The intracellular signaling networks regulated by the p75 neurotrophin receptor (p75^NTR^) substantially overlap with those linked to Aβ and to tau. Here we examine the hypothesis that modulation of p75^NTR^ will suppress the generation of multiple potentially pathogenic tau species and related signaling to protect dendritic spines and processes from Aβ-induced injury. In neurons exposed to oligomeric Aβ in vitro and APP mutant mouse models, modulation of p75^NTR^ signaling using the small-molecule LM11A-31 was found to inhibit Aβ-associated degeneration of neurites and spines; and tau phosphorylation, cleavage, oligomerization and missorting. In line with these effects on tau, LM11A-31 inhibited excess activation of Fyn kinase and its targets, tau and NMDA-NR2B, and decreased Rho kinase signaling changes and downstream aberrant cofilin phosphorylation. In vitro studies with pseudohyperphosphorylated tau and constitutively active RhoA revealed that LM11A-31 likely acts principally upstream of tau phosphorylation, and has effects preventing spine loss both up and downstream of RhoA activation. These findings support the hypothesis that modulation of p75^NTR^ signaling inhibits a broad spectrum of Aβ-triggered, tau-related molecular pathology thereby contributing to synaptic resilience.

## Introduction

Degeneration of neurites, and synapses and spines in particular, are among the pathophysiological features that best correlate with loss of cognition in Alzheimer’s disease (AD)^1,2^. Clinical^3^ and mouse model^4^ studies, suggest that Aβ drives synaptic degeneration and failure, in large part, through tau-mediated mechanisms^5^. Moreover, longitudinal AD studies have identified subjects with significant amyloid accumulation, but with largely absent neurite/synapse degeneration and/or cognitive impairment (termed AD resilience)^2^ and this has further stimulated interest in the identification of mechanistic targets and therapeutic approaches that might prevent degeneration promoted by the Aβ-tau axis. Significant challenges to these efforts include the wide array of pathological tau molecular mechanisms triggered by Aβ, and additionally, the possibility that there are concomitant, tau-independent mechanisms through which Aβ promotes synaptic failure.

Molecular mechanisms relevant to the neurite and synapse degeneration triggered by amyloid beta (Aβ) and occurring in AD include: increased phosphorylation, misfolding, cleavage and oligomerization of tau^6,7^; missorting of pathological forms of tau into dendrites and spines promoting deleterious protein–protein interactions^8^; and aberrant cofilin-mediated actin modification within spines^9^. Upstream signaling changes occurring in AD and likely contributing to these processes include increased tau kinase activity^10^, increased caspase mediated tau cleavage^11–13^, and dysregulated Rho kinase activity^14–17^.

Signaling networks regulated by p75^NTR^ have a substantial overlap with AD-related degenerative networks^18–20^. Moreover, p75 is expressed by most neuronal populations involved in AD, including those affected in the earliest stages^21,22^. Given these findings, and that p75^NTR^ in its unliganded state or in the presence of its principal central nervous system ligands, pro-neurotrophins, enables or promotes neuronal degeneration^23^, we and others have tested the hypothesis that knockout of intact p75^NTR^ would inhibit Aβ-related degeneration. In in vitro studies, degeneration of mouse hippocampal neuron neurites triggered by Aβ oligomers was blocked in cultures of p75^NTR–/–^ hippocampal neurons^24,25^. Consistent with these in vitro studies, injection of Aβ oligomers into the hippocampus of p75^NTR–/–^ mice resulted in decreased degeneration of basal forebrain neurons relative to wildtype mice^24^ and crossing of p75^NTR–/–^ mice with two different APP mutant AD mouse models resulted in decreased neurite degeneration without a decrease in Aβ levels^25,26^. In clinical studies, polymorphisms involving the genes encoding p75^NTR^ ligands proNGF and proBDNF^27,28^ and its co-receptors sortilin and sorCS2^29,30^ have been found to be associated with AD. In addition, associations between genetic polymorphisms involving p75^NTR^ and AD have been identified^31,32^. These genetic findings implicate the p75^NTR^/ligand/co-receptor signaling module as a candidate contributor in the development of AD. At the protein level, proNGF is increased in brain tissue or cerebrospinal fluid derived from mild cognitive impairment (MCI) and AD subjects with significant correlations between the proNGF level and cognitive score^23,33,34^.

In prior studies, we developed small molecule ligands of p75^NTR^ that modulate its activity to downregulate degenerative and upregulate trophic signaling^20,35^. One of these, LM11A-31, has been found to block several p75^NTR^-linked pathophysiological mechanisms in the context of AD^19^. LM11A-31 blocks neuritic dystrophy induced by Aβ oligomers in vitro, and in APP mouse models inhibits excess tau phosphorylation and misfolding as well as decreasing neuritic dystrophy^19,36–38^.

To investigate the role of p75^NTR^ in tau-related mechanisms and AD resilience, we determined the effects of the p75^NTR^ modulator LM11A-31 on tau molecular pathologies and neurite, spine and synaptic degeneration triggered by Aβ. We found that the compound had unexpectedly broad effects on tau-related molecular mechanisms induced by Aβ, and substantially inhibited degeneration of neurites and spines. These findings support small molecule p75^NTR^-targeting strategies for conferring resilience in the context of Aβ accumulation.

## Results

### p75^NTR^ modulation mitigates Aβ-induced neuritic and spine neurite degeneration

**Figure 1:**
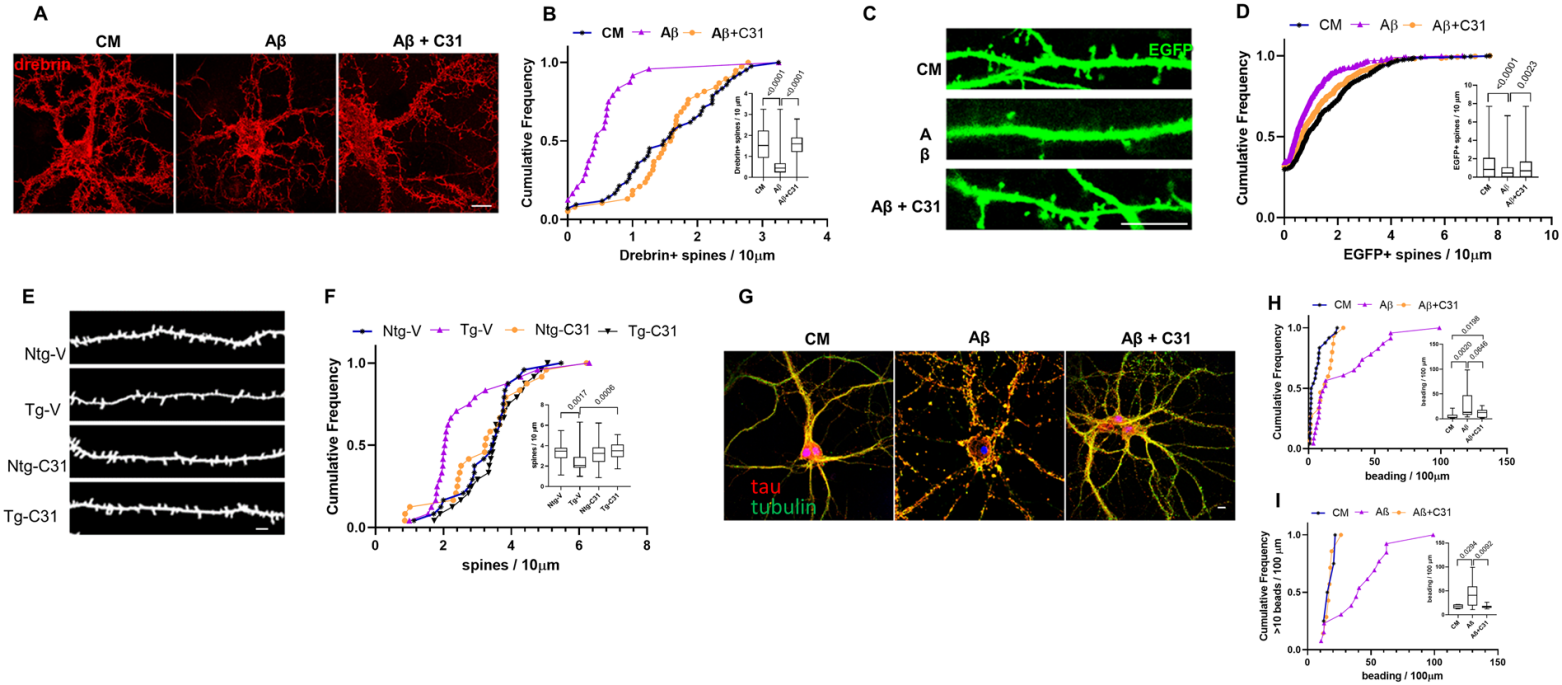
p75^NTR^ modulation mitigates Aβ-induced loss of spines and neurite injury. (**A–D**) 21 DIV hippocampal neurons were treated with CM, Aβ, or Aβ with 100 nM LM11A-31 and examined at 24 h following treatment. (**A**) Representative neurons stained for drebrin (red). (**B**) Quantitation of numbers of drebrin-positive spines per length of dendrite segment, displayed in cumulative frequency and (*inset*) box plots, with *p* values above the boxes from KS testing of indicated comparisons. n = 24–38 neurites per condition from a total of 3 independent experiments. (**C**) Dendritic spines of EGFP-transfected hippocampal neurons (green) were visualized at 24 h post-Aβ exposure. (**D**) Dendritic spine density quantitated and displayed as in (**B**). n = 36–43 neurons per condition from a total of 9 independent experiments. (**E**,**F**) 7.5–8.0-month-old non-transgenic mice (Ntg) and AβPP^L/S^ mice (Tg) were treated with either vehicle (V) or LM11A-31 (C31) by oral gavage for 3 months, at which time spine density of hippocampal CA1 neurons was determined in Golgi-stained dendrites using MBF Neurolucida. (**E**) Representative traced dendritic spine images. (**F**) Quantitative analysis of the dendritic spine density displayed as in (**B**). 3 neurons from each of 6 mice were analyzed from each treatment group (n = 18/group). Scale bars in each image, 10 µm. To assess neuritic injury, neurons were immunostained for tau (red) and tubulin (green) and discrete yellow regions (‘beads’), reflecting co-accumulation of tau and tubulin, were manually counted and expressed as number of beads/100 µm neurite beads. Scale bar, 10 µm (**G**). (**H**) Cumulative frequency analysis shows a population of higher-count segments in Aβ-treated cells not observed in the presence of C31 or without Aβ. *Inset* displays box plots with *p* values from KS testing for the indicated comparisons. (**I**) Analysis of distributions of high-count segments (> 10 beads/100 µm). n = 12–14 neurites/group derived from a total three independent experiments.

Hippocampal neurons at > 15 days in vitro (DIV) respond to addition of soluble Aβ oligomers with synaptic and neuritic degeneration rather than cell death, which occurs in less mature cultures^39,40^. Loss of structures containing drebrin, an F-actin binding protein present in dendritic spines, serves as a measure of Aβ-induced spine degeneration in vitro^39^ and studies have found that total drebrin levels are reduced in human AD and AD mouse model brain tissue, and drebrin-containing spines have been shown to be substantially reduced in the latter^41^. In the present study, exposure of 21 DIV hippocampal neurons to oligomeric Aβ caused an ~ 70% decrease in the median density and significant uniform left shift of the density distribution of drebrin-positive spines; this effect was blocked by LM11A-31 (Fig. 1A,B). These observations were further characterized by visualization and counting of spines, made visible by the transfection of cells with EGFP, which was previously utilized to assess Aβ effects on spines^42^. Here, spine densities in EGFP-expressing cells were decreased ~ 50% by Aβ with significant, though incomplete protection by LM11A-31 (Fig. 1C,D).

To determine whether p75^NTR^ modulation could affect final common pathways of AD-related spine loss in vivo, effects of LM11A-31 were determined in an APP-mutant AD mouse model, APP^L/S^, which exhibit amyloid plaques starting at age 3–4 months^43^ and reduced spine densities by age 7.0–7.5 months (*unpublished data, Longo Laboratory*). Mice were treated for 3 months with LM11A-31, from age 7.5–8 to 10.5–11 months, at which time tissue was harvested for assessment of hippocampal pyramidal neuron dendritic spines. Compared to wild-type mice, dendrites from APP-L/S mice exhibited a 42% reduction in spine density, while treatment of transgenic mice resulted in median spine densities and distributions matching those of vehicle-treated wild-type mice (Fig. 1E,F). LM11A-31 had no detectable effects on spine density in wild type mice. Since spine density is decreased in these mice at age 7.0–7.5 months when treatment with LM11A-31 began, these findings indicated that LM11A-31 treatment may shift the dynamics of spine formation and regression back towards normal. Notably, prior studies in the APP-L/S model demonstrated that similar treatments with LM11A-31 had no effect on Aβ levels measured by either ELISA or plaque quantitation^36,37^.

To determine the effects the of p75^NTR^ modulation on Aβ-induced dendritic degeneration, we examined neurite beading, a process linked with tau pathology involving disruption of the microtubule cytoskeleton along with localized accumulation of tau^44^. Exposure of 21 DIV hippocampal neurons to Aβ resulted in extensive neuritic beading, quantitated as number of beaded morphological profiles per 100 µm process length (Fig. 1G,H). Notably, though the median beading values were not significantly altered by LM11A-31, the distribution of beading across counted segments was significantly shifted towards a reduction in degeneration by the treatment, with a dramatic divergence between the distributions occurring above 10 beads/100 µm (Fig. 1I), with the treated cells trending similar to the control distribution. This pattern may be due to alterations in the kinetics of injury, but the sharp divergence may further suggest that LM11A-31 inhibits the progression to severe involvement, but not the initiation of tau-related Aβ-induced neuritic injury.

### Altering p75^NTR^ activities reduces AD-associated forms of tau induced by Aβ

**Figure 2:**
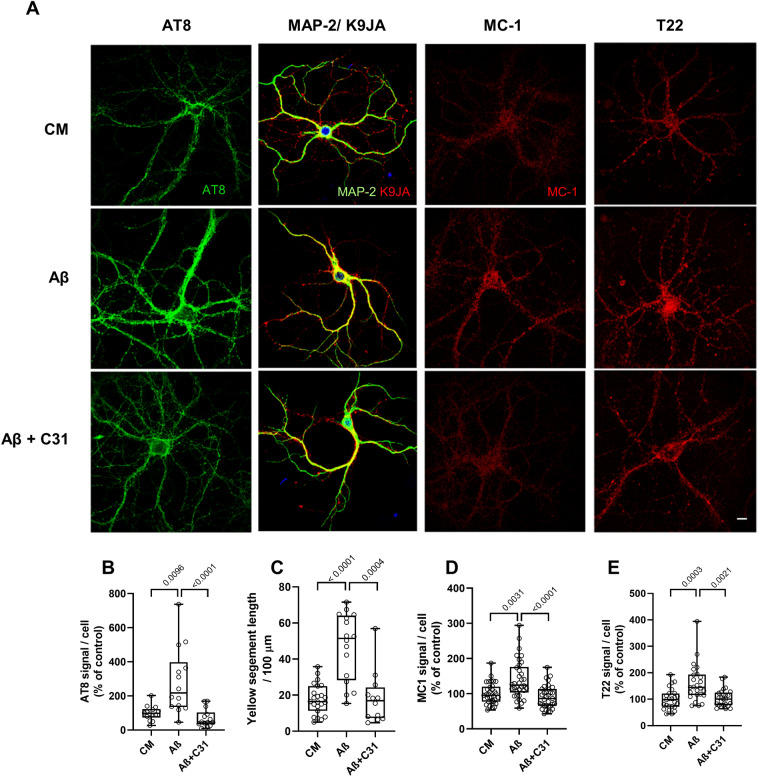
LM11A-31 prevents Aβ-induced tau phosphorylation, missorting, misfolding and oligomer accumulation. (**A**) Representative immunostaining for tau phosphorylation, missorting, misfolding and oligomer formation in hippocampal neurons. 21 DIV hippocampal neurons were treated with CM or Aβ ± LM11A-31 and immunostained with the indicated antibodies at 4 h for tau phosphorylation (AT8/green), 3 h for coincidence of microtubule-associated protein 2 (MAP2/green) tau and (K9JA/red) (missorting), 8 h for tau misfolding (MC-1/red) and 48 h for tau oligomerization (T22/red). Scale bar, 10 µm. (**B**–**E**) Total fluorescence intensity was measured and ratios of total fluorescence intensity to total cell numbers per field were calculated. (**B**) n = 14 neurons derived from 3 independent experiments. (**C**) n = 14–23 neurites derived from 3 independent experiments. (**D**) n = 30 neurons derived from 5 independent experiments. (**E**) n = 24 neurons derived from 4 independent experiments. Statistical significance of the indicated comparisons was determined in (**C**–**E**) using Kruskal–Wallis ANOVA with post-hoc Dunn’s multiple comparisons test and in (**B**) using Regular ANOVA with post-hoc Sidak’s multiple comparison testing.

Several tau modifications and configurations, including tau phosphorylation, cleavage, misfolding and missorting, have been proposed to mediate spine and neurite degeneration and dysfunction caused by Aβ^45–48^. Further, since LM11A-31 inhibited spine and neurite degeneration, and was previously shown to inhibit Aβ-triggered activation of tau kinases GSK3β and cdk5, as well as tau phosphorylation at Ser202^19^, it was of interest to determine whether one or more additional Aβ-induced tau alterations occurring in hippocampal neurons might be blocked by LM11A-31. Under these conditions, LM11A-31 suppressed Aβ-induced excess levels of Ser202 and Thr205 phosphorylation as reflected by AT8 binding^49^ (Fig. 2A *first column*, B). Increased phosphorylation at some sites on tau, including the AT8 sites, is associated with Aβ-provoked redistribution of tau from axons to the somatodendritic compartment^46^. Tau is predominantly located in axons under normal conditions (CM), while MAP2 is located in dendrites. In Aβ treated cells, tau is redistributed into dendrites, which can be quantified by measuring its colocalization with MAP2. LM11A-31 also inhibited this missorting of tau (Fig. 2A *second column*, C). The generation of misfolded tau isoforms which can be detected by antibodies such as MC-1, which recognizes an epitope formed by the apposition of two regions at each end of the tau peptide not normally in proximity, is associated with tau phosphorylation and the progression of AD^50,51^. Further, misfolding is associated with the production of oligmerized tau, considered to be a major toxic species^52^, which can be detected with the antibody T22^53^. Here, both MC-1 and T22 signals, which were increased by exposure to Aβ, were diminished by LM11A-31(Fig. 2A *third and fourth columns*, D,E). Though the co-dependencies and temporal sequences of the generation of these various tau alterations remain subjects of investigation, together these findings indicate that in this in vitro model of Aβ toxicity, LM11A-31 can function downstream of Aβ and broadly upstream of several known tau modifications associated with AD.

### Tau oligomerization and cleavage in APP^L/S^ mice is inhibited by modulation of p75^NTR^

**Figure 3:**
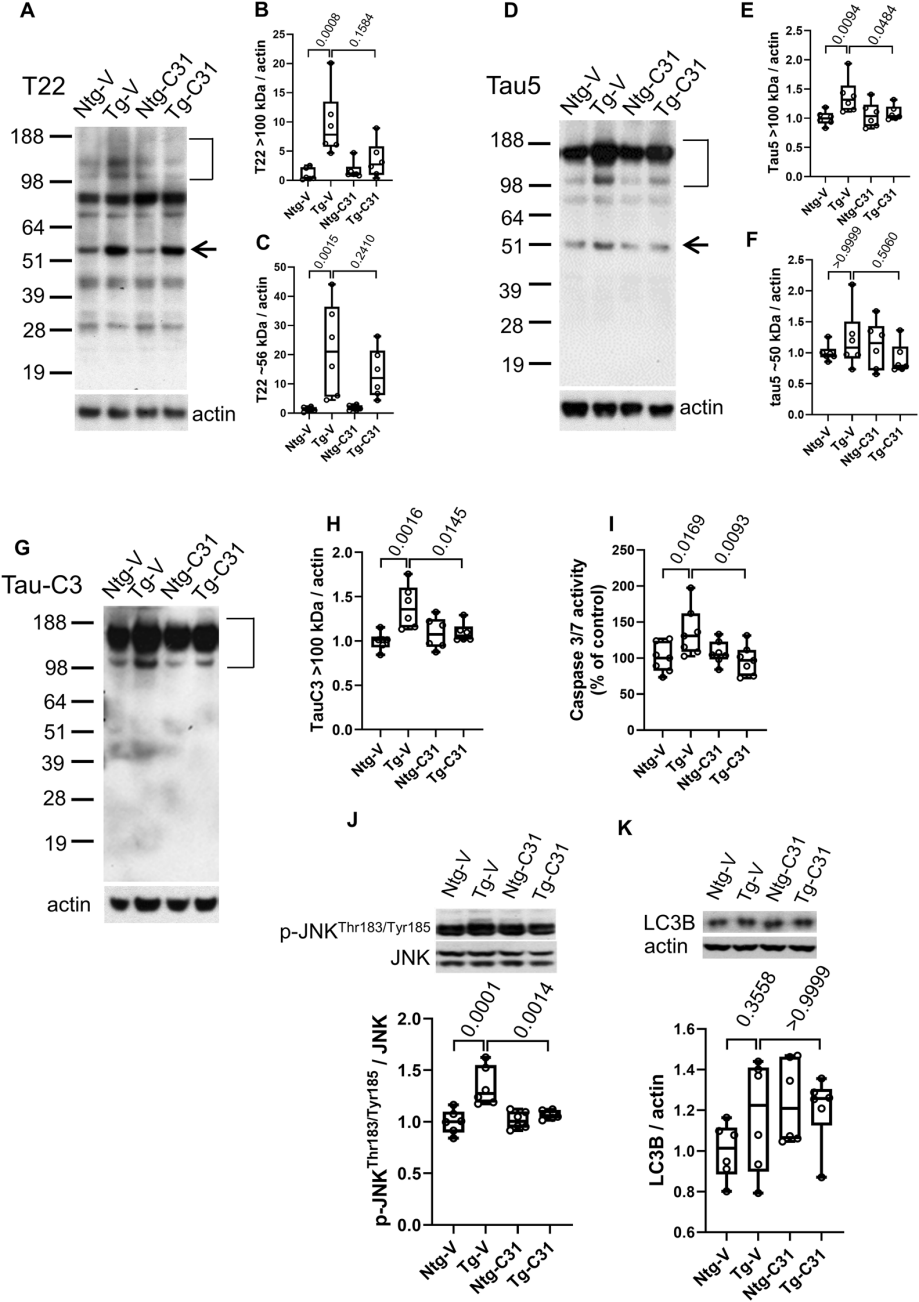
LM11A-31 decreases Aβ-induced tau oligomer accumulation, tau cleavage and injury signaling in vivo. 7.5–8.0-month-old non-transgenic mice (Ntg) and AβPP^L/S^ mice (Tg) were treated with LM11A-31 by oral gavage for 3 months. PBS extract fractions (for tau oligomers and cleavage) and RIPA extracts (for p-JNK^T183/Y185^, caspase 3/7 and LC3B) from hippocampal tissue were assessed by western blot or ELISA. For western blots, phospho-protein/total-protein or protein-of-interest/actin was determined as indicated; n = 6 mice per group, with three independent western blots averaged per animal. For caspase activity ELISA, n = 6 mice per group, with duplicate wells averaged per animal. (**A**) T22/oligomeric tau western blot. (**B**) Quantification of T22 signal > 100 kDa and (**C**) T22 ~ 56 kDa. (**D**) Tau 5 total tau western blot. (**E**) Quantification of Tau-5 (oligomer) signal > 100 kDa and (**F**) Tau-5 signal ~ 50 kDa (tau monomers). (**G**) Tau-C3 (cleaved tau) western blot. (**H**) Tau-C3 signal > 100 kDa. (**I**) Hippocampal extract caspase 3/7 activity normalized to Ntg-V. (**J**) Western blot analysis p-JNK/total JNK. **(K**) Western blot of LC3B/actin. Statistical significance of the indicated comparisons was determined in (**B**,**F**,**K**) using Kruskal–Wallis ANOVA with post-hoc Dunn’s multiple comparisons test and in (**C**,**E**,**H**,**I**,**J**) using Regular ANOVA with post-hoc Sidak’s multiple comparison testing.

Since tau oligomerization likely results from multiple tau metabolic mechanisms, including some which might be present in in vivo but not in in vitro models it was of further interest to determine the effects of modulating p75^NTR^ signaling on the accumulation of tau oligomers in vivo. Mutant APP-based mouse models do not demonstrate neurofibrillary tangles in the absence of human tau, but do form tau oligomers which can be detected with the T22 antibody on Western blots as high molecular weight bands in non-denaturing gels^53^. Treatment of mice with LM11A-31 resulted in a decrease in the elevated level of T22-binding oligomers > 100 kDa in PBS-soluble extracts present in AβPP^L/S^ mice relative to wildtype, though this did not reach significance (Fig. 3A,B). A ~ 56 kDa band, within the size range of either an oligomer of tau fragments, or alternatively, physiological murine tau^54^, was significantly increased in APP^L/S^ mice compared to non-transgenic mice (Fig. 3A,C) and though there was an apparent small decrease in this band associated with LM11A-31 exposure, this also did not reach significance. In addition, Tau5 antibody, which detects physiological as well as oligomeric tau forms^55^, was used to study aggregation. Tau5 detection of tau oligomeric species of ≥ 100 kDa was increased in vehicle treated APP^L/S^ mice compared to wild type but significantly reduced by LM11A-31 treatment (Fig. 3D,E). Of note, T22 and Tau-5 antibodies may recognize different, though overlapping, populations of tau oligomeric species^56^, derived from a diverse set of functionally distinct conformers^57^. Signal at ~ 50 kDa MW consistent with tau monomers remained unchanged between wild type and transgenic mice and LM11A-31 had no detectable effect (Fig. 3F).

Caspase-mediated tau cleavage at Asp421 has been proposed to contribute to tau toxicity^58^ and oligomer formation^59^, and cleaved tau is a component of tau oligomers and neurofibrillary tangles. Since Aβ can promote caspase activation^12^ and tau cleavage^59–61^, and given that p75^NTR^ has been linked to caspase activation^62^, it was of interest to examine the effects of p75^NTR^ modulation on accumulation of Asp421-truncated tau in APP^L/S^ mice. Using the tau-C3 antibody which is specific for tau cleaved at Asp421, western blot analysis demonstrated significantly increased levels of this product in APP^L/S^ mice which were largely abolished with LM11A-31 treatment (Fig. 3G,H). To assess potential mechanisms underlying this effect, caspase 3/7 activity, which is responsible for Asp421cleavage, was examined in hippocampal extracts using the Caspase-Glo 3/7 assay. Increased caspase3/7 activity was detected in APP^L/S^ mice compared to wild type mice and treatment with LM11A-31 reduced this activity to baseline levels (Fig. 3I). Since p75^NTR^ signaling may influence JNK activation, which in turn may lead to activation of caspases^62,63^, JNK activation, as indicated by the generation of phospho-JNK, was measured in these samples. p-JNK levels were increased in transgenic relative to wild type mice and this increase was prevented by LM11A-31 treatment (Fig. 3J).

While the above findings suggested that LM11A-31 might lead to reduced levels of tau oligomers by inhibiting their production, similar effects might occur due to promotion of tau degradation^64,65^. One major pathway regulating tau turnover and oligomer formation is autophagic activity, which may be reflected by levels of the autophagosome component, autophagy-related protein light chain 3 beta (LC3B). Treatment with LM11A-31 was not found to be associated with increased LC3B signal suggesting that it does not promote decreased oligomer levels through increased autophagic flux (Fig. 3K). Possible contributions of the ubiquitin–proteasome system remain to be assessed in this model. Together with the cell culture data, these in vivo findings provide evidence for several p75^NTR^/LM11A-31-associated mechanisms which may regulate the production of oligomeric tau isoforms. However, a significant contribution of increased degradation of those forms remains a possibility.

### Small molecule-mediated modification of p75^NTR^ signaling reduces Aβ-induced spino- and synaptotoxic Fyn kinase activities

**Figure 4:**
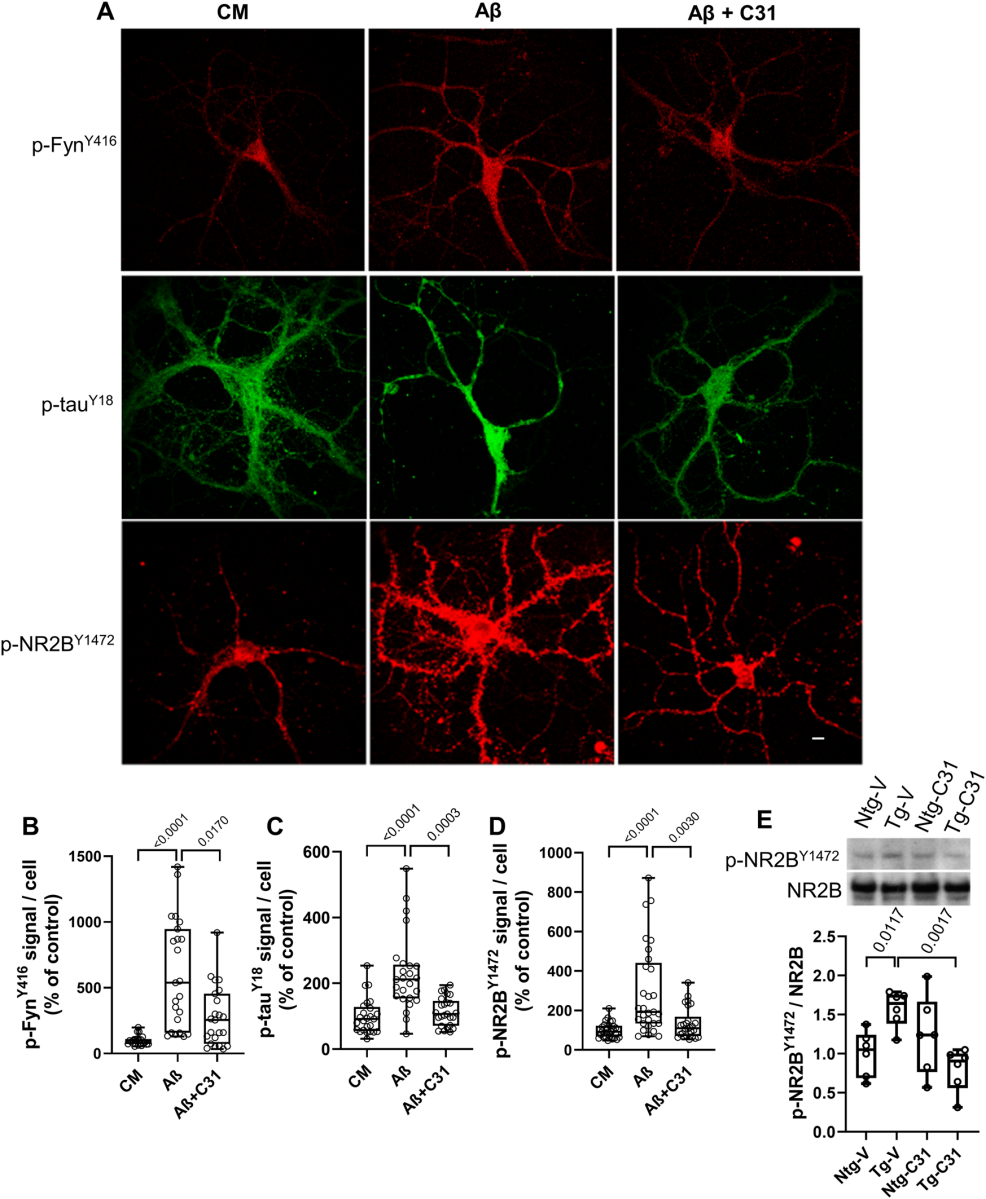
LM11A-31 modulates Aβ-induced Fyn activation and substrate phosphorylation. (**A**) 21 DIV hippocampal neurons were treated with CM or Aβ ± LM11A-31 and fixed and immunostained with the indicated antibodies after 20 min. Scale bar, 10 µm. (**B**–**D**) Total fluorescence intensity was measured and ratios of total fluorescence intensity to total cell numbers per field were calculated. For p-Fyn^Y416^, n = 18–22 fields derived from 4 independent experiments; p-NR2B^Y1472^, n = 24 fields derived from 6 independent experiments; p-tau^Y18^, n = 30 fields derived from 5 independent experiments. Statistical significance of the indicated comparisons was determined using Kruskal–Wallis ANOVA with post-hoc Dunn’s Multiple Comparisons test. (**E**) 7.5–8.0 months old non-transgenic mice (Ntg) and AβPPL/S mice (Tg) were treated with LM11A-31 by oral gavage for 3 months. RIPA protein extracts from hippocampal tissue were assessed by western blot analysis and the ratio of phospho-NR2B^Y1472^ to total NR2B protein was determined with normalization to Ntg-V. n = 6 mice per group, with three independent western blots averaged per animal. Statistical significance of the indicated comparisons was determined in (**C**,**D**) using Kruskal–Wallis ANOVA with post-hoc Dunn’s multiple comparisons test and in (**B**,**E**) using Regular ANOVA with post-hoc Sidak’s multiple comparison testing.

Aβ- and/or tau-related mechanisms may mediate synaptotoxicity through interactions with the protein kinase Fyn. Several lines of evidence suggest that increased missorting of tau and its accumulation in dendrites leads to increased binding to Fyn which facilitates Fyn-mediated phosphorylation of the NR2B subunit of NMDA receptors, which impairs their functions^4,66^. Since p75^NTR^ signaling has been implicated in activation of Fyn^67^ and LM11A-31 reduces tau missorting, it was hypothesized that LM11A-31 would inhibit excess Fyn phosphorylation/activation occurring in Aβ-treated neurons. In hippocampal neuron cultures, addition of Aβ led to increased p-Fyn^Y416^, indicative of Fyn activation^68^, and this was inhibited in the presence of LM11A-31 (Fig. 4A *first row*, B). Fyn kinase targets, which may play important roles in synaptotoxicity, include tau^Y18^ and NR2B^Y1472^, both associated with excitotoxic alterations in glutamate transmission^66,69,70^. Therefore, it was of interest to determine whether LM11A-31-mediated inhibition of Aβ-induced Fyn activation would inhibit accumulation of p-tau^Y18^ and p-NR2B^Y1472^. Treatment of DIV 21 hippocampal neurons with Aβ induced a significant increase in tau^Y18^ and NR2B^Y1472^ phosphorylation and these effects were significantly reduced by LM11A-31 (Fig. 4A *middle and bottom rows*, C,D). Similar results were obtained on in vivo analysis of p-NR2B^Y1472^ in APP^L/S^ hippocampal tissue (Fig. 4E). Together, these results are consistent with the possibility that LM11A-31 mediated suppression of Fyn activation contributes significantly to the overall effects of the compound on the inhibition of Aβ synaptic toxicity.

### Rho-family GTPase and cofilin responses to Aβ are mitigated by modulation of p75^NTR^ signaling

**Figure 5:**
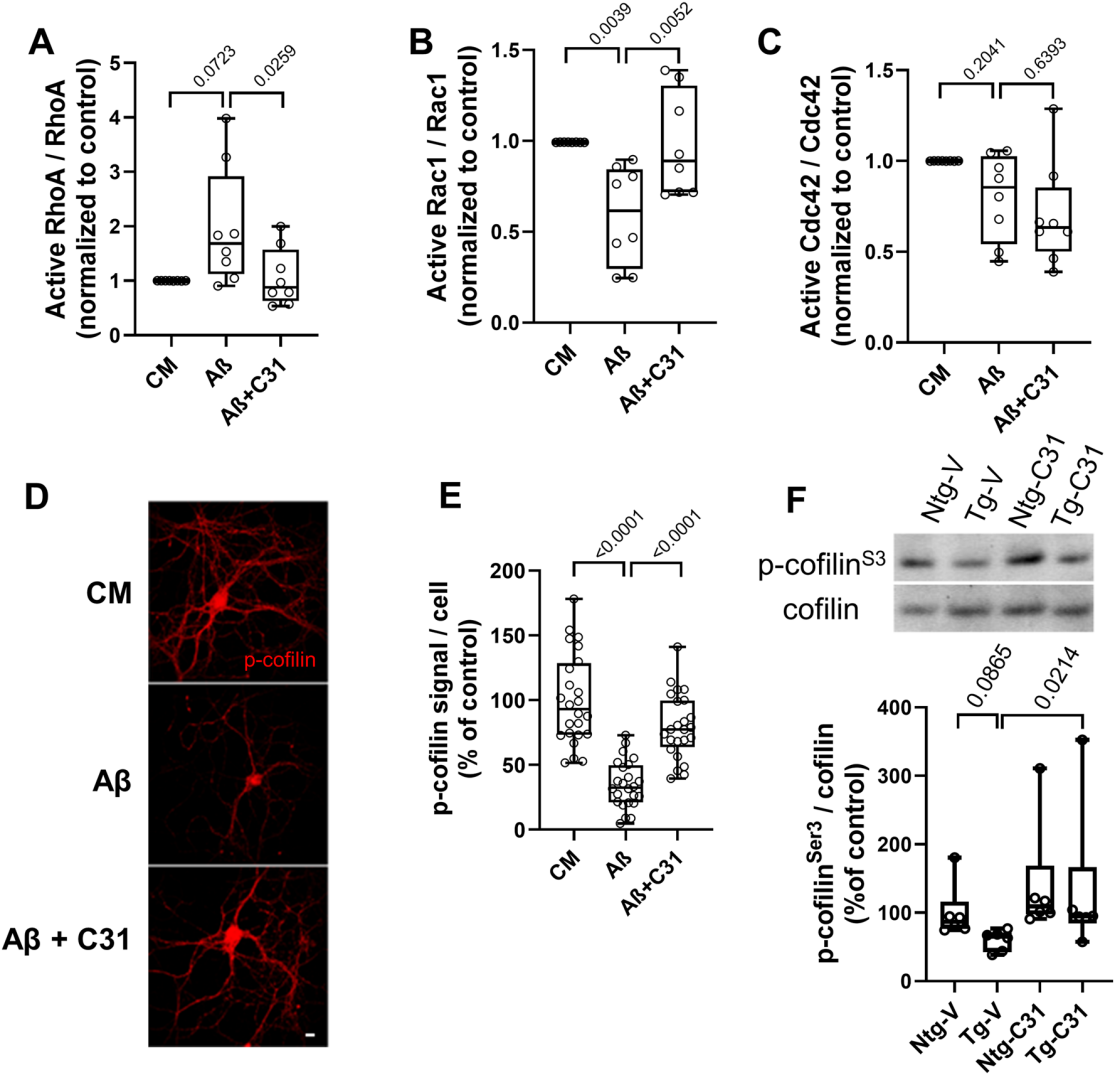
Aβ-induced changes in cofilin and Rho-family signaling are normalized by LM11A-31. (**A**–**E**) 21 DIV hippocampal neurons were treated with CM or Aβ ± LM11A-31 and cells were harvested after one hour for cell extract preparation. (**A**–**C**) For each assay measuring RhoA, Rac1 or Cdc42 GTPase, n = 9 protein preparations from independent experiments were measured, each protein preparation was tested using each of the three assays in duplicate and the two values were averaged. (**D**) Representative immunostaining images for p-cofilin^Ser3^. Scale bar, 10 µm. (**E**) For p-cofilin^S3^ quantitation, total fluorescence intensity over total cell number per field was calculated, n = 24 fields derived from a total of 4 independent experiments. Statistical significance was determined for the indicated comparisons using Kruskal–Wallis ANOVA with post-hoc Dunn’s Multiple Comparisons test. (**F**) 7.5–8.0-month-old non-transgenic mice (Ntg) and AβPPL/S mice (Tg) were treated with LM11A-31 by oral gavage for 3 months. RIPA protein extracts from hippocampal tissue were assessed by western blotting for the ratio of p-cofilin^Ser3^ to total cofilin signal. N = 6 mice per group, with the values from three independent western analyses averaged per animal. Statistical significance of the indicated comparisons was determined in (**A**,**B**,**C**,**F**) using Kruskal–Wallis ANOVA with post-hoc Dunn’s multiple comparisons test and in (**E**) using Regular ANOVA with post-hoc Sidak’s multiple comparison testing.

Rho family GTPases are important regulators of neurite growth and dendritic spine dynamics^71^. p75^NTR^ modulates RhoA activation and related actin depolymerization^72^ and Aβ can promote RhoA activation^73^ in vitro. Since LM11A-31 has been shown to inhibit pathologic excessive RhoA activation in a model of chemotherapeutic agent toxicity^74,75^, it was theorized that it might work similarly in Aβ toxicity models. Aβ exposure induced Rac1 inactivation in hippocampal neuron cultures and increased median RhoA activation, though the latter did not reach significance, and these effects were inhibited by LM11A-31 (Fig. 5A,B). There was no detectable change in Cdc42 activity nor response to LM11A-31 (Fig. 5C). An important downstream effector regulated by Rho GTPases, cofilin, is a highly conserved actin-binding protein, which plays an essential role in regulating actin filament dynamics and reorganization by stimulating the severing and depolymerization of actin filaments^76,77^. Aβ oligomer-induced spine loss and reduction of the spine protein drebrin has been linked to dephosphorylation at the cofilin Ser3 residue and resulting actin filament disassembly^9,78^. In the present study, cofilin phosphorylation at Ser3 was reduced by ~ 60% in hippocampal neurons treated with Aβ (Fig. 5D,E) and this reduction was inhibited by treatment with LM11A-31. Consistent with these in vitro results, and with observations in other AD animal models^9^, levels of cofilin phosphorylation were reduced in APP^L/S^ mice compared to vehicle treated-non-transgenic mice (Fig. 5F). Notably, these levels were restored by treatment with LM11A-31.

### Roles of tau phosphorylation and RhoA activation in the actions of LM11A-31 on Aβ-induced spine density loss

**Figure 6:**
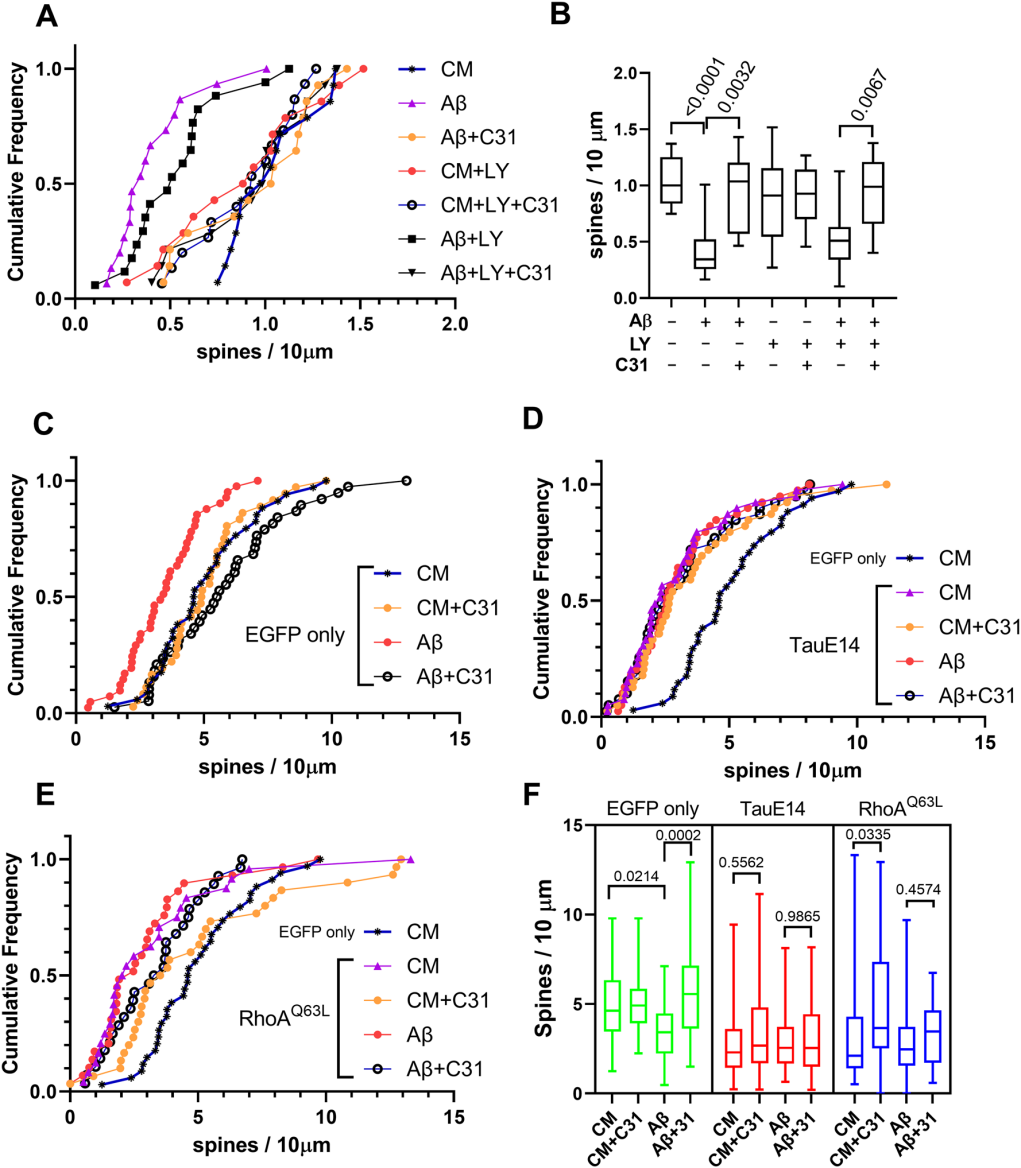
LM11A-31 inhibition of Aβ-induced spine loss is dependent on modulation of tau phosphorylation and RhoA activation. (**A**,**B**) 21 DIV hippocampal neurons were treated with CM or Aβ ± LM11A-31 with or without LY29002 (a PI3 kinase inhibitor), and examined at 24 h. The number of drebrin-positive spines per dendrite length was measured. (**A**) Cumulative frequency graph and (**B**) box plot of the indicated treatment groups. n = 14–16 fields derived from three independent experiments. *P* values for the indicated comparisons determined by KS testing. (**C**–**F**) DIV 20 hippocampal neurons were plasmid transfected for 2 h followed immediately by the addition of CM or Aβ ± LM11A-31. Dendritic spine density was determined after 24 h. (**C**) Cumulative frequencies of cells transfected with plasmid encoding enhanced green fluorescent protein (EGFP), (**D**) transfection with tau phosphomimetic, pRK5-EGFP-Tau E14, or (**E**) transfection with constitutively active RhoA, pcDNA3-EGFP-RhoA-Q63L. Distribution of EGFP/CM samples is included in graphs (**D**) and (**E**) as a reference. (**F**) Summary box plot of all groups. *P* values for the indicated comparisons determined by KS testing. n = 26–42 neurons for each condition derived from a total of 5 independent experiments.

In prior studies, LM11A-31 was shown to inhibit the Aβ-induced reduction of PI3K/AKT activation and increased c-JUN activation^19^, consistent with known roles of p75 in regulating PI3K/AKT and JNK signaling^20^. In the present study, addition of the PI3K inhibitor LY294002, previously found to substantially inhibit LM11A-31 support of immature neuron survival^35^, did not alter the effects of the Aβ oligomers on spines in hippocampal neuron cultures and did not inhibit LM11A-31-mediated restoration of spine densities (Fig. 6A,B). These findings suggest that the protective effect of LM11A-31 is not dependent on its activation of PI3K/AKT signaling.

To further examine the roles of the effects of LM11A-31 on the formation of pathogenic tau conformations and Rho-family GTPase activities in protection of dendritic spines, responses with transfection of mutant forms of tau and RhoA were determined. With an EGFP-only-expressing control plasmid, administration of LM11A-31 inhibited Aβ-induced spine loss (Fig. 6C) similar to the prior EGFP-spine observations (Fig. 1C,D). Preliminary spine density studies with transfection of EGFP-wild type tau found no differences with EGFP alone (Supplementary Figure 6), and the wild-type form was not further assessed. In 21 DIV hippocampal neurons transfected with tauE14 in which all 14 proline-directed serine/threonine phosphorylation sites are mutated to glutamate to emulate hyperphosphorylation^79^ or RhoAQ63L a constitutively active form of RhoA^80^, spine densities were significantly decreased (Fig. 6D–F). In cultures transfected with EGFP-tauE14, Aβ caused no additional decreases in spine density and LM11A-31 failed to prevent dendritic spine loss (Fig. 6D,F) either with or without Aβ. These observations provide further support for the idea that the protective effects of LM11A-31 are dependent, at least in part, on its inhibition of tau-mediated spinotoxicity. Since overexpression of wild-type tau does not reduce spines, the result with tauE14 is consistent with prevention of excess tau phosphorylation by LM11A-31 as a protective mechanism; however, effects on other tau-mediated mechanisms may also contribute. In addition, though the lack of further decline in spine density on adding Aβ is consistent with a principal role for pathogenic forms of tau in mediating Aβ-induced decreased spine densities, other Aβ-mediated mechanisms cannot be ruled out, as the effect achieved by tauE14 may represent a floor spine density value. RhoA-activating mutations such as that present in RhoAQ63Lmay increase the level and duration of RhoA activity after neuronal stimulation, effects similar to, but more pronounced than those occurring with wild-type RhoA overexpression^71^, and are also associated with decreased neuritic spine density^81^. Neurons transfected with RhoAQ63L, while attaining a reduction in spine density similar to that seen with tauE14 remained partially responsive to LM11A-31, suggesting that the compound may function parallel to or downstream of RhoA activation. In the presence of Aβ, there were no significant differences in distributions or median values with or without LM11A-31 (Fig. 6E,F) suggesting the possibility that the compound’s inhibition of Aβ spinotoxicity is dependent, in part, on its effects on RhoA. Whether this depends on the amount of RhoA, its localization, activity or another aspect of its physiology remains to be determined. These results suggest that to the extent that RhoA activity contributes to Aβ-mediated spinotoxicity, inhibition of RhoA activation may constitute one contributing factor to LM11A-31’s spinoprotective effects.

## Discussion

**Figure 7:**
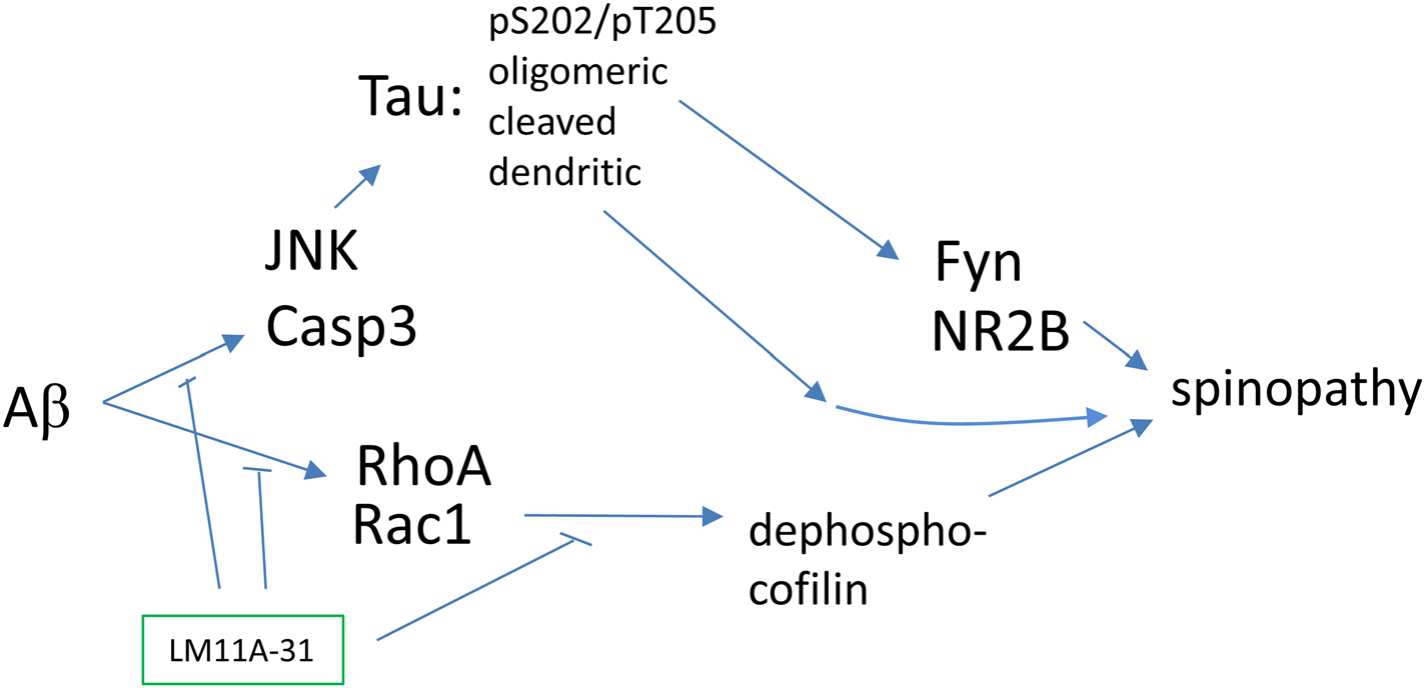
Model for LM11A-31/p75 modulation of Aβ-induced degenerative signaling. p75 signaling intersects several potentially degenerative Aβ-induced signaling pathways including those involving JNK, caspases and RhoA and that enhance formation of potentially toxic modified forms of tau. The present study points to regulation of tau phosphorylation and RhoA activation as two mechanisms promoted by modulation of p75 signaling likely to contribute to resilience of spine degeneration induced by Aβ.

Recent studies involving human subjects and multiple AD mouse models continue to support the hypothesis that Aβ triggers processes promoting neurite and synaptic degeneration through multiple molecular mechanisms, with several, though not all, involving tau-linked molecular pathology^82,83^. Given the likely limitations of single-mechanism approaches, a major goal is to develop therapeutic strategies capable of blocking as many such degenerative mechanisms as possible; including non-tau- as well as tau-based processes. Prior studies demonstrated that LM11A-31 inhibited Aβ-induced neuronal degeneration and that this effect was lost in cells expressing mutant p75^NTR^ lacking its extracellular ligand-binding domain or in the presence of nerve growth factor (NGF) acting as an LM11A-31 antagonist at p75^NTR^, suggesting that the protective effect is dependent on targeting p75^NTR^ signaling^19^. Prior work also demonstrated that LM11A-31 blocks Aβ-induced tau phosphorylation (AT8), activation of calpain, cdk5, c-jun, GSK3β, p38 kinase; and impairment of CREB and AKT activation^19,36,37^. The present study, focused on tau-related mechanisms, found that LM11A-31 prevents Aβ-induced and tau-linked pathologies including loss of dendritic spines and neurite degeneration, and identified an array of affected tau-related mechanisms through which this protective effect might occur. These include inhibition of tau phosphorylation, cleavage, misfolding, missorting and oligomerization – a novel and broad mechanistic profile in the context of existing AD therapeutic strategies under development. In addition to tau-based processes, LM11A-31 inhibited RhoA/Rac1/cofilin mechanisms that are also known to contribute to spine degeneration. Other effects detected in the APP^L/S^ mouse model included: inhibition of caspase-3/7 activation and tau cleavage, JNK activation, and excess NR2B phosphorylation. Taken together, these in vitro and in vivo findings establish a small molecule therapeutic model based on the inhibition of multiple key Aβ-related degenerative processes (Fig. 7).

The ability of LM11A-31 to inhibit accumulation and likely production of misfolded and oligomerized tau points to a new therapeutic category. Currently, two approaches based on direct targeting of tau and evaluated in preclinical studies have entered clinical trials^84^. One tau-centered method, is the application of tau antisense treatments, which is based on preclinical work showing reduced pathology in APP/Aβ-based tau KO mice^85^. Potential challenges to this approach include establishing the extent to which a therapeutic window exists in which a chronic reduction of tau does not significantly impair its critical physiologic functions yet substantially inhibits AD pathology. Moreover, the extent to which synaptic failure and degeneration in late onset AD is promoted by tau versus mechanisms unrelated to tau remains an open question. Another tau-based treatment approach is the administration of tau antibodies^84^. One challenge will involve the effective targeting of likely multiple toxic tau conformers/strains. Another is the possibility that tau toxicity might occur via tau structural intermediates and/or proximal/rapid interactions which are unlikely to be blocked by antibodies directed to the final toxic strain. Finally, limited antibody access within intracellular compartments where pathological forms of tau are likely generated might further impair this approach. The findings in the present study suggest that targeting multiple ‘upstream’ mechanisms affecting formation of pathogenic tau species, while maintaining levels of physiologic/non-oligomeric tau, is a tractable approach to protection against Aβ toxicity.

The principal approach to preventing tau hyperphosphorylation involves direct inhibition of individual tau kinases^86–89^. This may interfere with their physiologic functions and the possibility remains that the set of tau sites regulated by a given kinase would be insufficient to adequately prevent misfolding and toxic oligomer formation. Indeed, the extent to which small molecule tau kinase inhibitors can block tau misfolding and oligomer formation is not known. Moreover, whether any of these strategies prevent the degeneration of neurites, spines and/or synapses remains to be established.

Interactions of tau missorted into dendritic/spine compartments with the protein kinase Fyn is a proposed pathway by which tau mediates synaptotoxicity^4^. In this model, missorted tau mediates the ability of Fyn to cause excess phosphorylation of the NR2B subunit of the NMDA receptor^4,66,69,90^ and tyrosine residues in the proline-rich domain of tau, including Tyr18^91,92^. Excess phosphorylation of NR2B promotes increased recruitment of PSD95 into a synaptic protein complex resulting in excitotoxicity. The mechanisms of mislocalization are not fully established, but may involve phosphorylation as well as other post-translational modifications of tau, such as acetylation, not examined in this study, but also potentially influenced by LM11A-31^93,94^. Given the possible major role for tau missorting in promoting early stages of synaptic degeneration^46^, its correction by LM11A-31 may represent an important aspect of the compound’s spine- and neuritic process-protecting actions.

Truncated or cleaved tau has been proposed to contribute to cognitive impairment in AD^95,96^. Caspase-3- and calpain-mediated tau cleavage products have been detected in early stages of Aβ-induced neurotoxicity, including prior to tau hyperphosphorylation^59,97^ and neurite degeneration^98^. Caspase-3-truncated tau has been detected before the formation of NFTs and cell death^60^. Caspase-3 activation is elevated in AD^99^ and Aβ treatment of neurons induced activation of calpain and caspase-3/7 resulting in tau fragmentation^100^. Activated JNK, in addition to directly phosphorylating tau, can activate caspases via effects on mitochondrial apoptotic pathways^11^. Caspase-3 activity is increased in the dendritic spines of APP/Aβ transgenic mice and is associated with synaptic deficits^101^. Finally, genetic knockout and pharmacologic inhibition of caspase-3 rescue cognitive and synaptic deficits in an AD model and LTP deficits caused by soluble Aβ oligomers^101,102^. Thus, inhibition of JNK and caspase activation along with tau cleavage may each contribute to the protective effects of LM11A-31, representing yet another pathogenic pathway influenced by LM11A-31. Determination of the relative importance of these mechanisms awaits future studies.

The Rho subfamily of small GTPase proteins, which include RhoA, Rac1, Cdc42 and the actin-binding proteins such as cofilin and drebrin play significant roles in regulating actin cytoskeleton and spine dynamics^41,103,104^. In general, Rac1 and Cdc42 promote spine formation and maturation, whereas RhoA kinase activation is associated with spine retraction and synapse loss through its effects on actin cytoskeletal remodeling^105,106^. Aβ oligomers trigger a significant increase in RhoA activity in both SY5Y cells and in cultured hippocampal neurons^14,73,107,108^. Increased RhoA activity has been observed in the Tg2576 AD model, particularly in association with amyloid plaques^73^ and dystrophic neurites^109^. Studies in the hAPP J20 (Swedish and Indiana mutations) AD mouse model found a significant correlation between increased RhoA activity, dendritic spine loss and behavioral deficits^14^. A potential role for the Rho subfamily of small GTPase proteins in AD has also been uncovered in systems biology approaches^110^.

The state of cofilin phosphorylation modulates its regulation of actin cytoskeletal conformation, a key influence on synaptic spine status^111^. As noted, RhoA activity, which generally promotes phosphorylation and inactivation of cofilin via ROCK1 and LIMK is increased in AD, however increased cofilin activity has been observed in some AD brain and AD models, with decreased cofilin activation in others^9^. Recent detailed studies suggest that cofilin activation is spatially and temporally complex, with phosphorylation/inactivation occurring dynamically in post-synaptic densities, where it is associated with synaptic dysfunction and loss, and with dephosphorylated cofilin predominating in other cellular compartments^111^. Further, in another AD mouse model, phospho/inactive cofilin was found to decrease between 1 and 4 months of age, with a dramatic rise occurring by 10 months^9,112^. In addition, p21-activated kinase (PAK) has also been implicated as a cofilin kinase, with its activity regulated by p75^NTR^, possibly though modulation of Rac1^113^. The known p75^NTR^ modulation of Rho GTPase signaling and the findings here that LM11A-31 inhibits Aβ-driven effects on RhoA and Rac1 and alterations in cofilin activation and spine loss, and that the inhibition of Aβ-induced spine loss does not occur in the presence of constitutively active RhoA, illustrate another arm of the diverse effects promoted by LM11A-31/p75^NTR^ modulation which also have acute effects on synaptic transmission^19,114^.

Overall, the findings presented here demonstrate a broad set of Aβ pathogenic pathways influenced by LM11A-31/p75 receptor modulation, including inhibition of activation of multiple tau kinases along with reductions in Aβ-induced tau missorting, misfolding, cleavage and oligomerization. The effectiveness in reversing neurite/spine degeneration and lack of apparent deleterious effects indicate that LM11A-31, and more broadly, p75 modulation, may be an approach with significant therapeutic potential.

## Methods

### Materials

LM11A-31[2-amino-3-methyl-pentanoic acid (2-morpholin-4-yl-ethyl)-amide], was custom manufactured in the HCl salt form by Olon Ricerca Biosciences LLC (Concord, OH). Each preparation was purified by high-performance liquid chromatography (HPLC) and was greater than 99.8% pure. Structure was confirmed by nuclear magnetic resonance spectrometry and liquid chromatography/mass spectrometry. For use in vitro, LM11A-31 was dissolved in water prior to dilution in culture medium. For use in vivo, LM11A-31 was dissolved in water at a concentration of 5 mg/ml and stored at − 20 °C. Aβ_1–42_ peptide (HFIP form) was purchased from rPeptide (Bogart, GA). Aβ_1–42_ oligomers were prepared as described previously^19^. Other chemicals were purchased from Sigma-Aldrich Corp (St. Louis, MO), unless otherwise stated.

### Hippocampal neuron cultures and treatments

All animal procedures were conducted in accordance with the National Institutes of Health Guide for the Care and Use of Laboratory Animals using protocols approved by the Institutional Animal Care and Use Committee at Stanford University. Primary embryonic mouse hippocampal neuron cultures were prepared as previously described^19^. Briefly, neurons were plated on poly-D-lysine-coated (10 µg/ml) glass cover slips at 40,000–50,000 cells per well in 12-well plates (Corning Life Sciences, Tewksbury, MA). Cells were seeded in plating medium DMEM/F12 supplemented with 10% fetal bovine serum and 1 × penicillin/streptomycin (PS) for 2 h. Medium was then changed to Neurobasal medium (Invitrogen, Carlsbad, CA) supplemented with PS and B27 (Invitrogen) and 2 mM glutamine. After 21 days under these culture conditions, neurons demonstrate an adult-like phenotype with respect to neuronal polarity, microtubule and neurofilament cytoskeleton architecture, dendritic spines and functional synapses^40^. At 21 days cells were exposed to oligomeric Aβ at a concentration of 5 µM under conditions described in the “Results” and Figure Legends.

### Hippocampal neuron transfection studies

For assessment of dendritic spines, hippocampal neurons in vitro were transfected with a plasmid encoding enhanced green fluorescent protein (EGF; pEGFPN1, Clontech, Mountain View, CA). For analysis of p-tau and RhoA mechanisms, neurons were transfected with pRK5-EGFP-Tau E14 or constitutively active RhoA plasmid pcDNA3-EGFP-RhoA-Q63L (Addgene, Cambridge, MA). Transfections were performed over a 2-h period using Lipofectamine 2000 (EMD Millipore) at 20 days in vitro (DIV) as previously described^115^. Immediately following the 2-h transfection period, neurons were treated with CM, Aβ, or Aβ with or without concomitant addition of LM11A-31 (100 nM final concentration). After 24 h, neurons were fixed in 4% formaldehyde and slide-mounted with Pro-Long Gold (EMD Millipore). Images of EGFP transfected dendrites were acquired using a Leica DM5500 confocal microscope (Leica, Buffalo Grove, IL) with a 63 × objective. Spine density was measured by manual tracing using Neurolucida (MBF Biosciences, Williston, VT) and expressed as the number of spines per 10 µm of dendrite length using measurements obtained for multiple lengths of dendrite (n = number of lengths of dendrite) taken from 4–15 neurons per well across four or five independent neuronal cultures for each experimental condition. Counts were performed by an observer blinded to treatments.

### Immunofluorescence

Cultured hippocampal neurons were fixed in 4% formaldehyde for 20 min, permeabilized for 6 min in 80% ice-cold methanol, and incubated with primary antibodies at 4 °C overnight. Primary antibodies used were: rabbit polyclonal tau antibody K9JA (1:1000; DAKO, Carpinteria, CA) and mouse monoclonal MAP2 antibody AP20 (1:1000; Sigma, St. Louis, MO) to assess tau missorting; mouse monoclonal phospho-tau antibody AT8 (1:500; Thermo Fisher Scientific, Waltham, MA) and rabbit polyclonal T22 (1:500; EMD Millipore) to measure tau phosphorylation and tau oligomer formation; rabbit polyclonal synaptophysin antibody (1:300; Abcam, Cambridge, MA); MC-1 antibody which detects misfolded murine tau^116^; rabbit polyclonal tau antibody K9JA and mouse monoclonal α-tubulin antibody (1:1000; Sigma) to visualize neuritic beading. Other antibodies used for immunofluorescence included: mouse monoclonal drebrin antibody (1:300; Enzo, Farmingdale, NY), rabbit polyclonal p-cofilin^S3^ antibody (1:300; Abcam), rabbit polyclonal p-Fyn^Y416^ antibody (1:300; Cell Signaling, Beverly, MA); rabbit polyclonal p-NR2B^Y1472^ antibody (1:600; Sigma) and mouse monoclonal p-tau^Y18^ antibody (1:300; Medimabs, Montréal, QC). Secondary antibodies consisted of either donkey anti-rabbit or anti-mouse conjugated with either FITC or Cy3 (1:400; Jackson ImmunoResearch Laboratories, Inc. West Grove, PA).

### G-LISA Rac1, cdc42 and RhoA activation assays

For analysis of Rac1, cdc42 and RhoA activation, Cytoskeleton, Inc. (Denver, CO) G-LISA Rac1, cdc42 and RhoA Activation Assay Biochem Kits were used according to the manufacturer’s instructions. Briefly, hippocampal protein lysates were incubated in Rac1-GTP, RhoA-GTP or cdc42-GTP affinity plates for 30 min. Bound, activated Rac1, cdc42 and RhoA were measured with Rac1, cdc42 or RhoA-specific antibodies as activated/total signal from the same cell lysates.

### Caspase-3/7 activity assay

Caspase activity was measured using the luminescent Caspase-Glo 3/7 Assay, which reports both caspase 3 and 7 activity, according to manufacturer’s instructions (Promega, Madison, WI). One microgram of mouse hippocampal lysate in 25 µL of protein lysis buffer, plus 25 µL of Caspase-Glo 3/7 Reagent were added to each well of a white-walled 96-well plate, followed by incubation for 1 h at 37 °C. Sample luminescence was determined using a SpectraMax/M5 plate-reading luminometer (Molecular Devices, Sunnyvale, CA) and activity was expressed in relative luminescence units (RLU). Measurements were normalized as a percentage of the culture medium control mean value.

### Confocal imaging, quantification and data analysis

Cell culture images were collected by systematic field imaging in a blinded manner using a 63 × objective lens with a Leica DM5500 Confocal microscope. For quantification of pixel fluorescence intensity, UN-Scan-it Gel & Graph Digitizing Software Ver. 6.14 (Silk Scientific Inc, Orem, UT) was used. Signal per cell averages were calculated as the total level of fluorescent antibody-associated signal per field (p-tau AT8^S202/205^, synaptophysin, PSD95, T22, p-tau^Y18^, p-cofilin^S3^, p-Fyn^Y416^ or p-NR2B^Y1472^) divided by the total number of neurons in the field, and were normalized as a percentage of the culture medium control mean value. For quantification of neurite degeneration, Neurolucida (MBF Biosciences) in the manual mode was used to measure the total length of tubulin-positive neurites and the number of tau-positive beads, roughly circular to ovoid swellings arrayed along processes, in each field to yield the average number of tau-positive beads per 100-μm length of neurite for the given set of fields (3–6 fields per well). For quantification of tau missorting, Neurolucida was used manually to measure the total length of MAP2-positive neurites (green) and the length of neurite segments with MAP-2/tau co-localization (yellow) within a field, and the of co-localization length per 100-μm length of MAP2-positive neurites determined. All quantifications were performed in a blinded manner.

### AD mouse models, drug treatment, tissue harvesting and neuritic spine analysis

The AD mouse model utilized was the transgenic line Thy-1 hAPP 41B C57BL/6 which expresses the human amyloid precursor protein (APP) 751 (hAPP751) containing the London (V717I) and Swedish (K670M/N671L) mutations under the murine Thy1 promoter (APP-L/S)^43^. Male APP-L/S mice demonstrate progressive plaque deposition in the cerebral cortex and hippocampus beginning at 3–4 months of age^43^. One brain hemisphere was processed for modified Golgi staining and spine density measurements of hippocampal pyramidal neurons using established protocols, and the hippocampus of the other hemisphere was isolated for quantitative Western blotting as previously described^115,117^. Densities of dendritic spines of CA1 pyramidal neurons of the dorsal hippocampus were determined by tracing and marking Golgi-stained hippocampal neurons manually using Neurolucida while adjusting focus at 100X. Minimally overlapping neurons in the same region were selected. All branches from one dendritic tree of each neuron were traced and 3 neurons were analyzed per mouse.

### Protein extraction and Western blot analysis

Protein extracts were prepared by homogenizing frozen hippocampal tissue in RIPA lysis buffer (150 mM NaCl; 1% NP-40; 50 mM Tris, pH7.4; 1 mM EDTA; 10% glyceryl, 1 mM PMSF) with Pierce Protease and Phosphatase Inhibitor Tablets (Cat# A32959, ThermoFisher, Waltham, MA)^118^. Tau oligomer fractions were prepared by homogenizing frozen hippocampal tissue in phosphate buffered saline with a protease inhibitor (Cat#A32955, ThermoFisher) and 0.02% NaN3 using a 1:3 (w/v) dilution. Preparations were centrifuged at 10,000 × *g* for 10 min at 4 °C. Protein concentration was measured using the Precision Red Advanced Protein Assay (Cytoskeleton, Inc). Supernatants were aliquoted and stored at − 80 °C until use^55^. For Western blotting, 20–40 µg of total protein from each sample was run per lane on precast NuPAGE 4–12% Bis-Tris Gels for SDS-PAGE (ThermoFisher) then transferred to PVDF membranes. After blocking with 5% nonfat dried milk at room temperature for 1 h, membranes were probed overnight at 4 °C with one of the following antibodies: T22 (1:1000, EMD Millipore); cleaved-tau-Asp421 (1:10,000, EMD Millipore); Tau 5 (1:5000; BioLegend, San Diego, CA); p-JNK^T183/Y185^ (1:1000, Cell Signaling); JNK (1:1000, Cell Signaling); NR2B (1:1000); p-Fyn^Y416^ (1:1000, Cell Signaling); Fyn (1:2000; Santa Cruz Biotechnology, Santa Cruz, CA); p-NR2B^Y1472^ (1:1000, BD BioScience, San Jose, CA); actin (1:10,000; Sigma) or L3B (1:3000; Novus Biologicals, LLC, Littleton, CO). Secondary antibodies were either horseradish peroxidase (HRP)-conjugated anti-rabbit IgG (1:10,000; ThermoFisher) or anti-mouse IgG (1:10,000; DAKO) ECL (GE Healthcare, Sunnyvale, CA). Immunoreactive band densities were measured using Un-Scan-It gel software (Ver. 6.14, Silk Scientific Inc).

### Statistical analyses

Graphpad Prism 8 and Microsoft Excel were used for statistical data analysis, with α set at *p* < 0.05 for all tests. Box-plot graphs display the median (middle line), 25th and 75th percentiles (extent of the box) and range (whiskers) derived from at least three independent experiments. Data normality was determined for each set with the Shapiro–Wilk test. Non-parametric testing with Kruskal–Wallis ANOVA and Dunn’s post-hoc analysis was used when normality testing was not passed, otherwise regular ANOVA with Sidak’s multiple comparison post-hoc testing was utilized for group comparisons as indicated. Outcomes of in vivo experiments included in post-hoc testing were; first, differences between vehicle and drug in the transgenic context; and second, as a measure of quality control, differences between wild type and transgenic mice. Sample distributions were compared with two-sample Kolmogorov–Smirnov (KS) testing.

### Ethical approval and informed consent

All animal procedures were conducted in accordance with the National Institutes of Health Guide for the Care and Use of Laboratory Animals using protocols approved by the Institutional Animal Care and Use Committee at Stanford University.

## Supplementary information

Supplementary Information.

## Data Availability

All data generated or analyzed during this study are included in this published article.

## Abbreviations

AD: Alzheimer’s disease
Aβ: Amyloid beta
APP: Amyloid precursor protein
NMDA: *N*-Methyl-D-aspartate receptor
NTR: Neurotrophin receptor
NGF: Nerve growth factor
EGFP: Enhanced green fluorescent protein
PI3K: Phosphatidylinositol 3-kinase
p: Phosphorylated
Rac: Ras-related C3 botulinum toxin substrate 1
RhoA: Small GTPase RhoA
Fyn: Fyn kinase
KS testing: Kolmogorov–Smirnov testing
