# Supplementary material for: Small molecule modulation of the p75 neurotrophin receptor inhibits multiple amyloid beta-induced tau pathologies
Source: Sci Rep. 2020 Nov 23;10:20322. doi: 10.1038/s41598-020-77210-y (PMC7683564; doi:10.1038/s41598-020-77210-y)

Small molecule modulation of the p75 neurotrophin receptor inhibits multiple amyloid beta-induced tau pathologies

Tao Yang[a], Kevin C. Tran[a,1], Anne Y. Zeng[a,1], Stephen M. Massa[b*], and Frank M. Longo[a*]

[a]Department of Neurology and Neurological Sciences, Stanford University School of Medicine, Stanford, CA 94305
[b]Department of Neurology, San Francisco Veterans Affairs Medical Center, and Dept. of Neurology, University of California, San Francisco, San Francisco, CA, 94121

Supplementary Data: Full length western gel images and tau transfection

Supplementary Figure 1

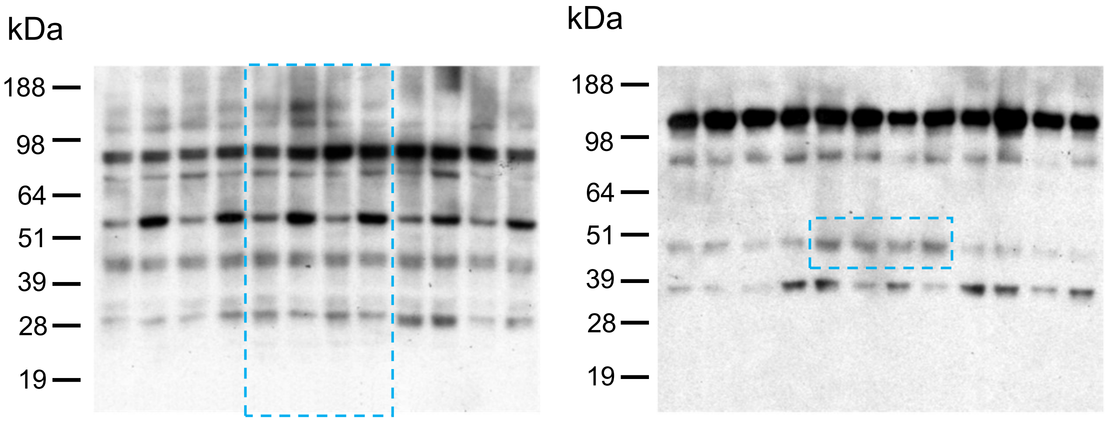

T22 **actin**

Supplementary Figure 2

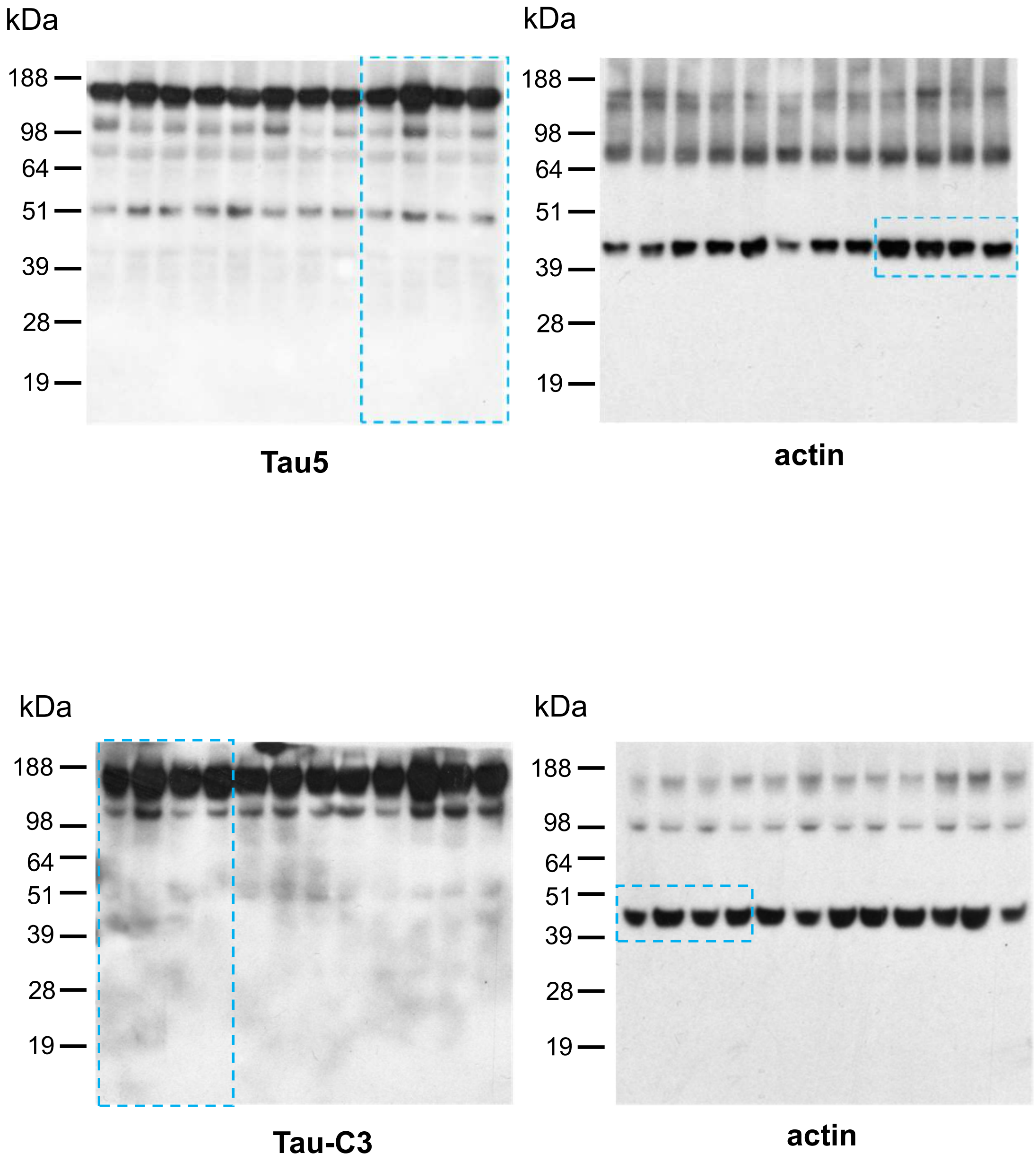

Supplementary Figure 3

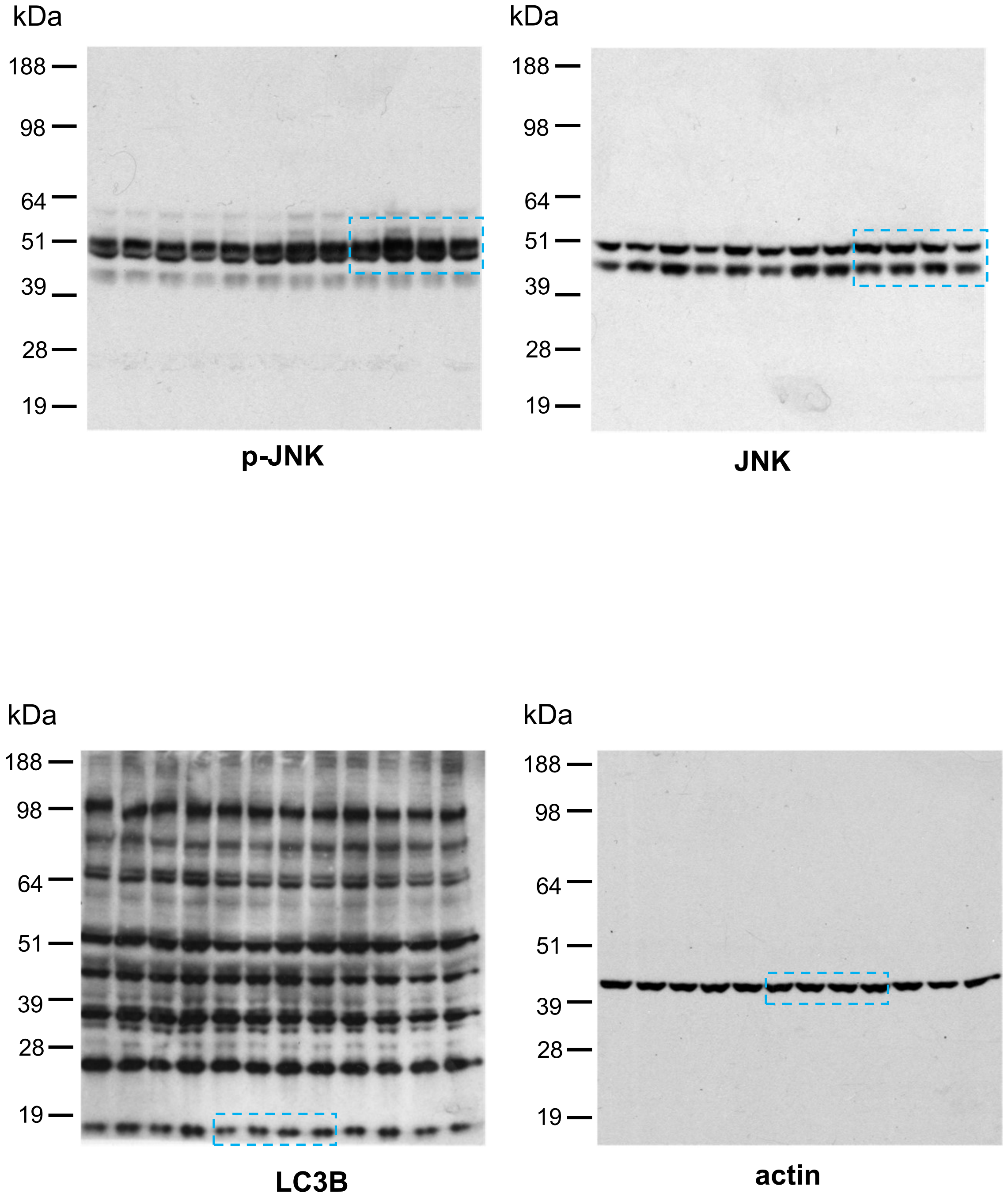

Supplementary Figure 4

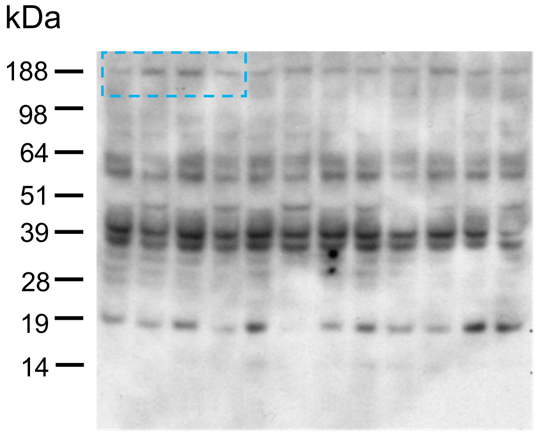

p-NR2B

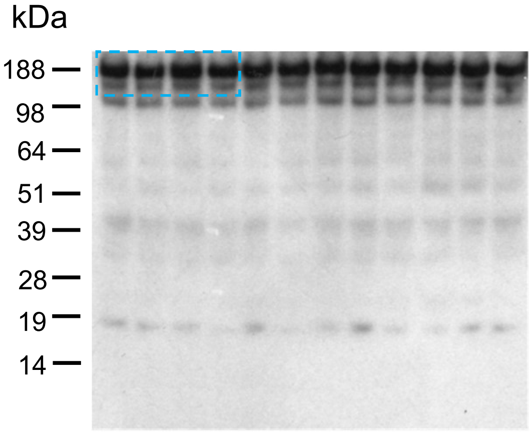

NR2B

Supplementary Figure 5

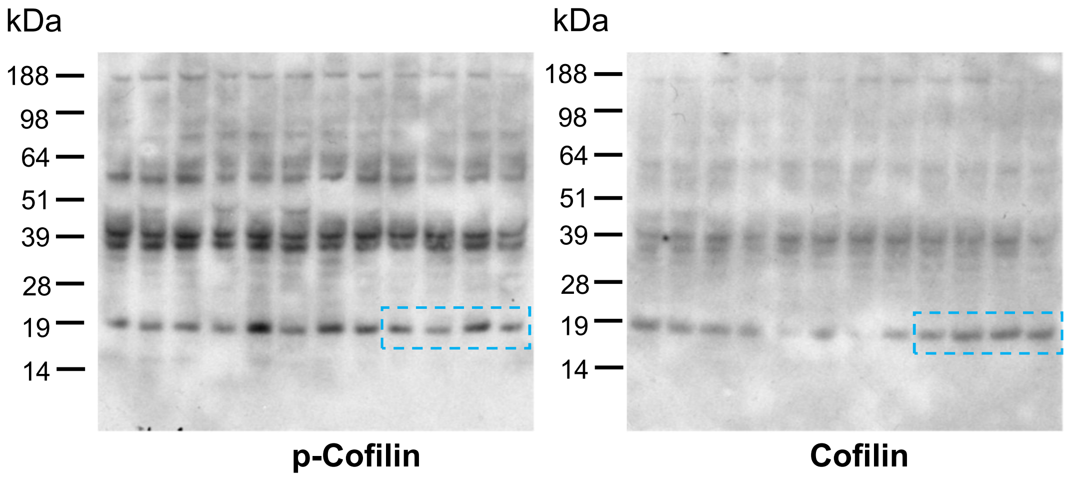

Supplementary Figure 6

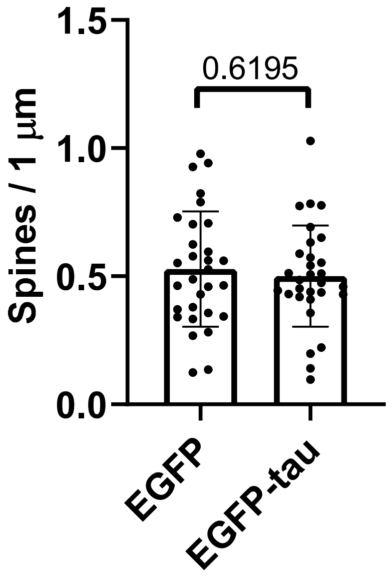

Supplement: Supplementary file 1 — Supplementary Information. [file 41598_2020_77210_MOESM1_ESM.pdf]
